# Effect of Phacoemulsification on Intraocular Pressure in Eyes with Functioning Tube Shunts

**DOI:** 10.18502/jovr.v18i2.13180

**Published:** 2023-04-19

**Authors:** Wesam Shamseldin Shalaby, Sonali Patel, Sophia S. Lam, Allen Ganjei, Aakriti Garg Shukla, Natasha Kolomeyer, Daniel Lee, L. Jay Katz, Marlene R. Moster, Jonathan Myers, Reza Razeghinejad

**Affiliations:** ^1^Wills Eye Hospital, Glaucoma Research Center, Philadelphia, PA, USA; ^2^Tanta Medical School, Tanta University, Tanta, Gharbia, Egypt; ^3^Sidney Kimmel Medical College, Thomas Jefferson University, Philadelphia, PA, USA; ^4^College of Medicine, Drexel University, Philadelphia, PA, USA

**Keywords:** Cataract Extraction, Glaucoma Drainage Implants, Intraocular Pressure, Phacoemulsification

## Abstract

**Purpose:**

To evaluate the effect of phacoemulsification on intraocular pressure (IOP) in eyes with functioning tube shunts.

**Methods:**

This was a retrospective chart review of primary open-angle glaucoma (POAG) patients with a functioning tube who underwent phacoemulsification and had ≥ 24 months of follow-up. The primary end point was defined as surgical failure (IOP > 21 mmHg) at month 24, progression to no light perception (NLP) vision, glaucoma reoperation, or implant removal. Surgical failure defined as IOP > 18 and > 15 mmHg, changes in visual acuity (VA), IOP, and number of medications were assessed.

**Results:**

Twenty-seven eyes of 27 patients with moderate or severe POAG were included. The mean age of the patients was 64.2 ± 10.8 years. The interval between the tube shunt and phacoemulsification was 28.8 ± 25.0 months. At the end of the study, four (14.8%) eyes met the failure criteria; the average time to failure was 9.3 ± 3.8 months. The causes of failure were high IOP in two (50.0%) and glaucoma reoperation in two (50.0%) eyes; however, no eyes progressed to NLP vision. Surgical failure defined as IOP > 18 and > 15 mmHg showed an increasing failure rate (18.5% and 48.5%, respectively).Themean IOP and medications number remained stable at month 24 compared to baseline (*P* = 0.131 and *P* = 0.302, respectively). Initially, VA showed improvement, with the greatest improvement at 6 months (*P* = 0.001), but at 24 months the improvement was no longer significant (*P* = 0.430).

**Conclusion:**

Phacoemulsification in patients with functioning tubes did not change the mean IOP in most of the patients (86.2%); the number of medications also did not increase.

##  INTRODUCTION

Cataract surgery in patients with preexisting glaucoma or glaucoma surgery is challenging considering the potential impairment of postoperative intraocular pressure (IOP) control. The development of cataract after glaucoma surgery is not uncommon.^[1]^ It has been suggested that intraocular inflammation during and after cataract surgery may result in scarring and fibrosis of the filtering trabeculectomy blebs.^[2]^ Similarly, the inflammatory cells and mediators may cause fibrosis around the shunt reservoir.^[3]^ However, there is no definite evidence supporting this theory. Several studies have investigated the effect of cataract surgery on trabeculectomy blebs. There have been mixed results on the bleb survival. While some studies showed increased failure of trabeculectomy and IOP elevation, ${}^{\left[4--7\right]}$ others reported no effect. ${}^{\left[8--10\right]}$ The literature on the effect of cataract extraction in eyes with prior tube shunts is limited.^[3,11,12]^

Prior studies showed lack of change in IOP and medications number in functioning tubes following phacoemulsification; however, they had a limited sample size, variable follow-up duration, and heterogenous type of glaucoma.^[3,11,12]^ The study aimed to investigate the survival of tube shunts after phacoemulsification in patients with primary open-angle glaucoma (POAG).

##  METHODS

This single-center retrospective case series was performed at a tertiary eye care center. The study protocol was reviewed and approved by the Institutional Review Board of Wills Eye Hospital, Philadelphia, PA, United States; and the approval number was [19-857]. The study was conducted in accordance with the Health Insurance Portability and Accountability Act regulations. The study protocol adhered to the tenets of the Declaration of Helsinki and the guidelines for human studies. As this was a retrospective study with de-identified data, informed consent was not required. The medical records of consecutive patients diagnosed with POAG who were treated with tube shunts including the Ahmed glaucoma valve (AGV; New World Medical Inc., Rancho Cucamonga, CA, USA) or the Baerveldt glaucoma implant (BGI; Advanced Medical Optics, Santa Ana, CA, USA) followed by phacoemulsification between 2008 and 2018 were reviewed.

Patients aged 18 years or older who had undergone successful tube shunt surgery for POAG with IOP ≤ 21 mmHg, with or without medications, followed by phacoemulsification were included. Exclusion criteria were angle closure, neovascular, or inflammatory glaucoma, cataract extraction by any technique other than phacoemulsification, phacoemulsification combined with any glaucoma surgery, or a follow-up of less than two years after phacoemulsification. In patients with both eyes meeting the inclusion criteria, only the first eye was included.

Visits at baseline, postoperative day 1, week 1, months 1, 3, 6, 12, 18, and 24 after the cataract surgery were reviewed. Demographic data such as age and sex, and medical and surgical history were collected. Preoperative clinical data included best-corrected visual acuity (BCVA), IOP, topical glaucoma medications, and cup/disc ratio (CDR) at three consecutive visits prior to phacoemulsification. Postoperative data included BCVA, IOP, glaucoma medications, CDR, postoperative complications, and need for additional glaucoma surgery.

The primary outcome measure was the cumulative rate of surgical failure at 24 months. Surgical failure was defined as IOP > 21 mmHg with medications at two consecutive visits, progression to no light perception (NLP) vision, and glaucoma reoperation including second tube shunts, cyclophotocoagulation, or removal of the existing tube shunt. Changes in VA, IOP, glaucoma medications, and CDR at month 24 were compared to baseline. Additionally, analyses of surgical failure defined as IOP > 15 and > 18 mmHg were performed.

### Statistical Analysis 

Statistical analyses were performed using the SPSS software version 27.0 (IBM Analytics, Chicago, IL, USA). Snellen VA measurements were converted to logarithm of the minimum angle of resolution (logMAR) equivalents for the purpose of data analysis. Continuous variables were presented as mean ± standard deviation. Proportions (%) were used to describe categorical variables. Paired sample *t*-tests were used to compare continuous variables within the same group. *P*-values < 0.05 were considered as significant. Kaplan–Meier survival analysis with log-rank tests were used to assess the survival.

##  RESULTS

Twenty-seven eyes of 27 patients were included in this study (16 AGV and 11 BGI). Baseline patient characteristics are shown in Table 1. The mean age was 64.2 ± 10.8 years, and 16 (59.3%) patients were females. All patients had moderate to severe glaucoma. Six patients (22.2%) had prior trabeculectomy before the tube shunt surgery. The mean LogMAR VA, IOP, and number of glaucoma medications were 0.94 ± 0.92, 16.0 ± 4.7 mmHg, and 2.4 ± 1.5, respectively. The average interval between tube shunt surgery and phacoemulsification was 28.8 ± 25.0 months.

**Figure 1:**
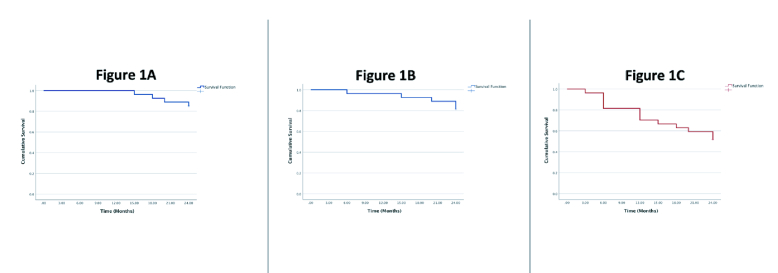
Kaplan–Meier Survival Plot of cumulative probability of surgical failure in patients with phacoemulsification and prior tube shunt surgery. Surgical failure was defined as intraocular pressure > 21 mmHg (1A), > 18 mmHg (1B), or > 15 mmHg (1C).

At month 24, a total of four (14.8%) eyes met the failure criteria. Failure occurred at 19.3 ± 3.8 months after cataract extraction. Reasons for failure were high IOP in two (50.0%) eyes and glaucoma reoperation in two (50.0%) others. No eye progressed to NLP vision. Higher failure rate was observed with the surgical success defined as IOP < 18 and < 15 mmHg (18.*5*% and 48.5%, respectively). The rate, reasons, and time to failure with the three failure criteria are shown in Table 2. Kaplan–Meier survival analysis showing the cumulative rate of surgical failure at 24 months using the three failure criteria is displayed in Figure 1.

Visual acuity improved after surgery and the difference with baseline was statistically significant at month six, with an improvement in logMAR VA of 0.41 ± 0.54 units (*P* = 0.001), but not at month 24 (0.11 ± 0.67-unit improvement, *P* = 0.430). No eyes progressed to NLP vision.

**Figure 2:**
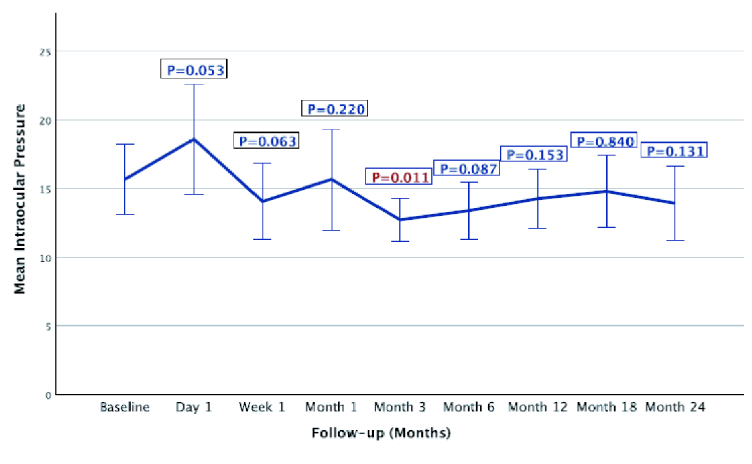
Intraocular pressure changes over time in patients with phacoemulsification and prior tube shunt surgery. Intraocular pressure remained stable through two years following phacoemulsification. Significant reduction was observed at month three (*P* = 0.011).

Baseline and follow-up IOPs are presented in Figure 2.Patients who met the failure criteria due to reoperation for glaucoma implant removal or progression to NLP vision were censored in statistical analysis of the follow-up visits. At postoperative day one visit, IOP was higher compared to baseline (18.1 ± 6.2 mmHg vs 16.0 ± 4.7 mmHg, *P* = 0.053). Then, a tendency for lower IOP as compared to baseline was observed at all follow-up visits, but not statistically significant (*P* > 0.05), except in the third month at which IOP was significantly lower compared to baseline (13.0 ± 3.7 mmHg vs 16.0 ± 4.7 mmHg, *P* = 0.011). At month 24, the IOP was lower by 1.6 ± 5.1 mmHg compared to baseline (*P* = 0.131).

**Figure 3:**
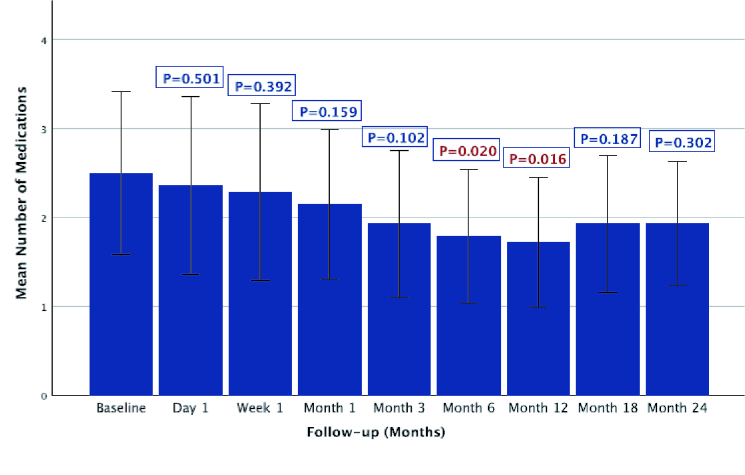
Medication number changes over time in patients with phacoemulsification and prior tube shunt surgery. Medication number remained stable through two years following phacoemulsification. Significant reduction was observed at month six (P = 0.020) and month 12 (P = 0.016).

Figure 3 shows the number of glaucoma medications at baseline and follow-up. A decremental trend for the number of glaucoma medications was observed at all time points; however, it was statistically significant at postoperative months 6 and 12 compared to baseline (*P* = 0.02 and *P* = 0.016, respectively). At month 24, the mean number of medications was 0.3 ± 1.5 lower compared to the baseline (*P* = 0.302).

Hyphema (1 [3.7%]), IOP spikes (IOP elevation ≥ 10 mmHg from the baseline) (1 [3.7%]), and inflammatory reaction ( > +1 cells in the anterior chamber) (14 [51.9%]) were the early postoperative complications; all resolved with conservative management. No eyes developed persistent corneal edema. During the follow-up period, one eye (3.7%) required tube revision and two (7.4%) eyes received second tube shunt.

**Table 1:** Baseline Characteristics of Patients with Phacoemulsification and Prior Tube Shunt Surgery.


Number of eyes	27
Number of patients	27
Age, yr	64.2 ± 10.8
Sex, Females: *N* (%)	16 (59.3)
Surgical eye, Right: *N* (%)	16 (59.3)
Baseline visual acuity: LogMAR	0.94 ± 0.92
Baseline intraocular pressure: mmHg	16.0 ± 4.7
Baseline medications number	2.4 ± 1.5
Baseline cup/disc ratio	0.7 ± 0.2
Glaucoma severity: *N* (%)	
Moderate	11 (40.7)
Severe	16 (59.3)
Glaucoma intervention prior to tube shunt surgery	
None	13 (48.1)
Selective laser trabeculoplasty	8 (29.6)
Trabeculectomy	6 (22.2)
Duration between tube shunt surgery and phacoemulsification: Months	28.8 ± 25.0
	
	

**Table 2:** Month 24 failure in patients with phacoemulsification and prior tube shunt surgery.


**Failure criteria 1: IOP > 21 mmHg**	
Failure: *N* (%)	4 (14.8)
Reasons of failure: *N* (%)	
IOP > 21 mmHg	2 (50.0)
Reoperation for glaucoma	2 (50.0)
Progression to NLP	0 (0.0)
Time to failure: Months	19.3 ± 3.8
Failure criteria 2: IOP > 18 mmHg	
Month 24 failure: N (%)	5 (18.5)
Reasons of failure: N (%)	
IOP > 18 mmHg	3 (60.0)
Reoperation for glaucoma	2 (40.0)
Time to failure: Months	17.8 ± 7.6
Failure criteria 3: IOP > 15 mmHg	
Month 24 failure: N (%)	13 (48.1)
Reasons of failure: N (%)	
IOP > 15 mmHg	11 (84.6)
Reoperation for glaucoma	2 (15.4)
Time to failure: Months	12.6 ± 7.2
	
	
white<bcol>2</ecol>IOP, intraocular pressure; NlP, no light perception

##  DISCUSSION

Our study shows that the mean IOP and number of glaucoma medications remained stable for two years following cataract extraction in POAG patients with a functional tube shunt. The rate of surgical failure was 14.8%, 18.5%, and 48.5% at IOPs of > 21, < 18, and < 15 mmHg, respectively. While the effect of cataract surgery on the trabeculectomy function has been heavily studied,^[4,5,6,7,8,9,10]^ there are limited studies on the tube shunts, and all have included various forms of open-angle glaucoma.^[3,11,12]^

Gujral et al^[12]^ retrospectively investigated the outcomes of phacoemulsification in eyes with functioning AGVs. The study included 23 eyes of 19 patients with an average follow-up of 1.6 ± 0.6 years after the cataract surgery. Similar to our study, the mean IOP and number of medications did not change after phacoemulsification (*P* > 0.05 for both). However, the follow-up in our study was longer. The pattern of postoperative IOP change was similar to our study; increased from 14.5 ± 3 mmHg (preoperative) to 19.2 ± 6.3 mmHg on postoperative day one, declined at month one, and remained stable afterward. The surgical failure defined as IOP > 21 mmHg was observed in two (9%) eyes and one of them (4.0%) underwent second tube shunt. These findings were comparable to our failure (14.8%) and reoperation (7.4%) rates. On the other hand, more eyes experienced IOP spikes at day one postoperatively (17.0%) compared to ours (3.7%).

Erie et al^[3]^ retrospectively studied the effects of phacoemulsification on nine eyes of eight patients with a functioning BGI with an average follow-up of 21.0 ± 3.0 months. The mean IOP and number of medications did not change significantly at the last follow-up visit (*P* = 0.830 and *P* = 0.170, respectively). Only one eye required second glaucoma surgery.

In another retrospective study, 11 eyes of 11 patients with double plate Molteno shunt (8), single plate Molteno shubt (2), or BGI (1) were followed for 21.0 ± 27.0 months following cataract surgery.^[11]^ Phacoemulsification was performed in eight eyes and extracapsular cataract extraction was done in three eyes. No significant difference was observed between the mean IOP and the number of medications before and after cataract extraction (*P* = 0.850 and *P* = 0.440, respectively). Three eyes had IOP > 21 mmHg and only one of them required glaucoma surgery.

Although VA improved in the first six months, the vision was not statistically different from baseline at month 24. This is consistent with prior studies, although in some studies it was attributed to corneal edema.^[11,12]^ Corneal edema was not observed in our study. Posterior capsule opacification could be a potential cause for the lack of change in vision after cataract extraction.

The high IOP at day one may be related to the postoperative period IOP spikes following cataract surgery due to viscoelastic agents.^[13,14]^ The IOP decline afterward could be because of the IOP-lowering effect of phacoemulsification in glaucomatous eyes.^[15,16]^ The mechanism is not clear, but may be related to widening of the anterior chamber angle, decreased aqueous production by the ciliary body due to capsular bag contraction, or increased aqueous outflow due to trabecular meshwork stretching.^[15,16][17]^

The results of our study are in line with other studies on IOP profile following cataract extraction in patients with a functional tube, but the results on trabeculectomy are controversial. Some studies showed that phacoemulsification increased the surgical failure of trabeculectomy,^[4,5,6,7]^ and others found no effect on bleb survival.^[8,9,10]^ The mechanisms of IOP elevation following cataract surgery in those with a functional filtering surgery (trabeculectomy or tube shunts) seems to be similar. Inflammatory cells and cytokines released following cataract surgery may cause scarring of the filtering bleb.^[2,11]^

The average duration between tube shunt surgery and phacoemulsification in this study was variable (28.8 ± 25.0 months). It is known that the time interval between the two surgeries may have prognostic ramifications. It is recommended to postpone the cataract surgery following filtering surgery as long as possible, without compromising patient's quality of life. A six-month gap between filtering surgery and the subsequent cataract extraction increases the chances of bleb survival.^[18]^

The patients in our study had phacoemulsification 28.8 months after shunt surgery and were followed for 24 months (about five years after shunt surgery). The failure rate in these patients was comparable with the rate reported in Ahmed Baerveldt Comparison (ABC) and Ahmed versus Baerveldt (AVB) studies.^[19,20]^ These findings confirm that phacoemulsification, at least, had no negative impact on the survival of tube shunts. However, such comparisons cannot conclude that phacoemulsification may augment the IOP-lowering effect of tube shunts, giving the different follow-up duration and the inclusion of secondary and refractory glaucoma types in the ABC and AVB studies.^[19,20]^

Our study has several limitations. There were no preset criteria for the addition of medications or preforming another surgery and has been at the discretions of surgeons. This is the universal issue with all retrospective studies. Additionally, the sample size was small as only POAG patients with a complete two-year follow-up were included. The follow-up period in our study was longer than prior studies and contrary to them we included only one eye of each patient and only POAG cases. Compared to all prior studies we had the largest sample size (27 vs 9, 11, and 23 eyes), and all patients were followed-up for at least for 24 months.^[3,11,12]^ Furthermore, both valved (AGV) and non-valved (BGI) tubes were included in this study which makes the results closer to the real-world practice.^[19,20]^

In summary, the current study showed that POAG patients with functioning tube shunts can maintain IOP control after phacoemulsification, suggesting that cataract surgery may not have a negative impact on the survival of tube shunts.

##  Financial Support and Sponsorship

None.

##  Conflicts of Interest

None.
